# Causal relationship between hepatic function indicators and thrombocytopenia risk in early-stage hepatitis B virus infection: evidence from clinical observational studies and mendelian randomization analyses

**DOI:** 10.3389/fimmu.2025.1440317

**Published:** 2025-05-29

**Authors:** Tian-bin Chen, Jian-wei Jiang, Hong-yan Guo, Xiao-tong Chen, Shuai Zhi, Yu-hai Hu, Ya Fu, Yong-bing Zeng, Can Liu, Qi-shui Ou, Shi-tao Rao

**Affiliations:** ^1^ The First Affiliated Hospital of Fujian Medical University, Fujian Key Laboratory of Laboratory Medicine, School of Medical Technology and Engineering, Fujian Medical University, Fuzhou, China; ^2^ Department of Laboratory Medicine, National Regional Medical Center, Fujian Medical University, Fuzhou, China; ^3^ Department of Bioinformatics, Fujian Key Laboratory of Medical Bioinformatics, Institute of Precision Medicine, School of Medical Technology and Engineering, Fujian Medical University, Fuzhou, China; ^4^ The School of Public Health, Fujian Medical University, Fuzhou, China; ^5^ Department of Hepatopancreatobiliary Surgery, The First Affiliated Hospital of Fujian Medical University, Fuzhou, China; ^6^ School of Biomedical Sciences, The Chinese University of Hong Kong, Hong Kong, China

**Keywords:** HBV: hepatitis B virus, MR: mendelian randomization, TPO: thrombopoietin, HCV: hepatitis C virus, snps, single-nucleotide polymorphisms, IV: instrumental variable, RCT: randomized controlled trial

## Abstract

**Background:**

Thrombocytopenia is a common occurrence in patients with hepatitis B virus (HBV) infection, particularly in those with liver cirrhosis. However, it can also manifest in the early stages of HBV infection, before the onset of liver cirrhosis. Despite its prevalence, the molecular mechanisms underlying thrombocytopenia in this context are not well understood. Therefore, the primary aim of this study was to investigate whether common hepatic function indicators have a significant causal role in this mechanism.

**Methods:**

We conducted a retrospective examination of the association between HBV infection and thrombocytopenia risk in apparently healthy participants who underwent health screening examinations. Subsequently, we investigated the causal relationship between multiple hepatic function indicators and thrombocytopenia risk by integrating clinical observational studies and univariate/multivariate Mendelian randomization (MR) analyses.

**Results:**

Among 16,464 participants who underwent health screening examinations, 2,730 subjects (16.58%) tested positive for HBsAg. The prevalence of thrombocytopenia was significantly higher in HBsAg-positive subjects compared to healthy controls (*P*<0.001). Univariate and stepwise multivariate logistic regression analyses identified lower albumin and higher alanine aminotransferase (ALT), alkaline phosphatase, and total bilirubin levels as independent factors significantly associated with thrombocytopenia risk (OR=1.95~6.60). Univariate and multivariate MR analyses further confirmed that ALT had significant causal effects on thrombocytopenia risk (adjusted *P*<0.05). Notably, we also observed significant trends of a higher prevalence of thrombocytopenia with elevated ALT levels in both the clinical raw and propensity score matching cohorts (*P*=0.015 and 0.014, respectively).

**Conclusions:**

This study identified multiple hepatic function indicators as independent factors associated with thrombocytopenia risk. Notably, our findings provided the first dual confirmation of the causal effect of the injury indicator ALT on thrombocytopenia risk, as evidenced by both clinical observational studies and genetics-based MR analyses, prior to the development of liver cirrhosis.

## Highlights

- The prevalence of thrombocytopenia was significantly higher in HBsAg-positive subjects compared to healthy controls in a clinical observational study involving 16,464 participants who underwent health screening examinations.
- Our univariate and multivariate logistic regression analyses revealed that lower ALB and higher ALT, ALP, and TBIL were independent factors significantly associated with an increased risk of thrombocytopenia.
- Our findings presented the first dual confirmation of the causal effect of the injury indicator ALT on thrombocytopenia risk, as evidenced by both clinical observational studies and genetics-based MR analyses, prior to the development of liver cirrhosis.

## Introduction

Chronic hepatitis B virus (HBV) infection is a global health concern and is the leading cause of cirrhosis and hepatocellular carcinoma (1). Furthermore, it can give rise to various extrahepatic manifestations, such as thrombocytopenia, which is strongly associated with increased bleeding complications, longer hospital stays, and higher mortality rates (2–5). The pathogenesis of thrombocytopenia in HBV infection primarily involves factors such as splenomegaly, hypersplenism, portal hypertension, cirrhosis, autoantibodies to platelets, virus-induced bone marrow suppression, and decreased thrombopoietin (TPO) production (6, 7). Nevertheless, there has been no observed correlation between free portal pressure and platelet count, and thrombocytopenia may persist even after splenectomy or portal decompression in certain cirrhotic patients (8, 9). Notably, thrombocytopenia has been observed in some HBV infection patients who have not yet progressed to liver cirrhosis or hypersplenism (10). Hence, the precise etiology of thrombocytopenia remains incompletely understood in HBV infection patients.

Although the relationship between thrombocytopenia and HBV or hepatitis C virus (HCV) infection is well-established in previous studies (10–12), the impact of common hepatic function indicators on the development risk of thrombocytopenia in early-stage HBV infection remains understudied. Elevated levels of ALP and hyperbilirubinemia have been identified as risk factors for thrombocytopenia in HBV infection patients (13). The TPO, which is synthesized in the liver, bone marrow, and kidney, plays a crucial role in regulating the development and maturation of megakaryocytes and the subsequent release of platelets (14). In HBV infection patients, the level and/or activity of TPO is decreased, contributing to the pathogenesis of thrombocytopenia (8). However, the causal correlations between liver function markers (such as liver enzymes or proteins) and the risk of thrombocytopenia, as well as the underlying molecular mechanisms, remain unclear.

Mendelian randomization (MR) utilized hundreds to thousands of single-nucleotide polymorphisms (SNPs) as instrumental variables (IVs) to deduce causal relationship between exposure and outcome traits. Due to the random distribution of alleles, the principle of MR could be likened to a randomized controlled trial (RCT), making it less susceptible to confounding factors and reverse causality (15, 16). Moreover, MR is based on summary statistics obtained from genome-wide association study (GWAS), which typically involve large sample sizes. This robust method allows for the inference of causality, particularly in cases where establishing causality through conventional observational studies is challenging (17–21).

In the present study, we conducted a retrospective analysis to investigate the potential association between HBV infection and the risk of thrombocytopenia in a large cohort of apparently healthy participants who underwent health screening at the First Affiliated Hospital of Fujian Medical University. Furthermore, we explored the causal relationship between common hepatic enzymes or proteins and the risk of thrombocytopenia by performing clinical observational studies with logistic regression analyses, as well as conducting univariate and multivariate MR analyses using publicly available GWAS summary statistics. Additionally, we assessed the prevalence of thrombocytopenia across multiple stratified levels of the potential causal factors in this large cohort. The findings from this study could contribute to the etiology of thrombocytopenia in the early stages of HBV infection.

## Materials and methods

### Design and participants for clinical observational studies

A flow chart of the participants included in the present study was shown in **Supplementary Figure 1**. The present study retrospectively analyzed participants who had undergone comprehensive health screening at the physical examination center of the First Affiliated Hospital of Fujian Medical University (Fuzhou, China) from January 2021 to July 2022. Prior to enrollment, all participants underwent ultrasonography examination. The exclusion criteria for the study included co-infection with hepatitis A, C, and/or D virus; autoimmune hepatitis; alcoholic liver disease; non-alcoholic fatty liver disease; splenomegaly; hypersplenism; portal hypertension; a history of therapy with anti-platelet drugs, immune inhibitors, or hepatotoxic drugs; a history of any tumor type; incomplete data. Since liver biopsy is an invasive procedure and not suitable for all participants undergoing health examination, the FIB-4 statistic (FIB-4 = (Age × AST)/(PLT × ALT^1/2^)) was used to assess the fibrosis stage. Participants with a FIB-4 value of ≥3.25 were considered to have a 97% specificity and a positive predictive value of 65% for advanced fibrosis (Ishak fibrosis score 4-6, indicating early bridging fibrosis to cirrhosis) (22–26). Additionally, participants with ultrasonography results indicating liver fibrosis or cirrhosis were also excluded if their FIB-4 value was above 3.25.

The initial stage of this study involved enrolling 19,142 participants from Chinese Han population. However, a total of 2,678 participants were excluded based on specific criteria. These criteria included age ≤16 years (n=311), age ≥60 years (n=2250), FIB4 ≥3.25 (n=42), and seropositive for both HBV surface antigen (HBsAg) and HBV surface antibody (HBsAb) (n=75). Ultimately, the study included 16,464 participants, among whom 2,730 subjects were diagnosed with HBV infection based on their serum HBsAg being positive for more than 6 months. Thrombocytopenia was defined as a platelet count below 150×10^9^/L, according to the National Cancer standard (http://ctep.cancer.gov) (5, 10, 12).

Among the 2,730 subjects who tested positive for HBsAg, only 131 individuals (4.80%) were diagnosed with thrombocytopenia, and these individuals tended to be older (47.04 ± 8.43 *vs.* 42.71 ± 9.15, *P*<0.001) (**Supplementary Table 1, Table S1**). To adjust for baseline age and reduce the impact of selection bias between the thrombocytopenia and non-thrombocytopenia groups, propensity score matching (PSM) was employed using the ‘matchlist’ package in the R programming language. Patients were matched in a ratio of 1:5 that randomly based on the logit of the PSM, utilizing the greedy algorithm and the Nearest Neighbor Matching method. Subsequently, the univariate and stepwise multivariate logistic regression analyses were conducted to identify independent risk factors for thrombocytopenia in HBV infection patients. The strength of association was expressed using odds ratios (OR) with 95% confidence intervals (CI). Statistical significance was set at a p-value below 0.05. Ethical approval for this study was granted by the Ethics Committee of the First Affiliated Hospital of Fujian Medical University.

### Measurement of laboratory data

The levels of HBsAg, HBsAb, HBeAg, HBeAb, and HBcAb were measured using commercially available enzyme-linked immunosorbent assay (ELISA) kits (Beijing Wantai Ltd., China). The presence of HBsAg indicated HBV infection, while HBsAg-negative participants were considered as healthy controls. Biochemical tests, such as alanine aminotransferase (ALT) or aspartate aminotransferase (AST), were analyzed using an automated analyzer (Cobas 8000, Roche, Germany). Abnormal or normal values for liver enzymes or proteins were determined based on the widely used reference interval (27, 28). Whole blood cell counts, including white blood cells (WBC), neutrophils (NEU), lymphocyte (LYMPH), monocytes (MONO), red blood cell (RBC), hemoglobin (Hb), and platelet (PLT), were measured using an automated analyzer (Siemens ADVIA 2120, Germany) in blood samples anticoagulated with EDTA-2K.

### Statistical analyses in clinical observational study

Continuous variables that presented as mean ± standard deviation were compared using the Student’s t-test or the Mann-Whitney U test. Statistical differences of categorical variables were calculated using the chi-square test or Fisher’s exact test. Chi-square for trend between variables and subgroups were performed by the Cochran-Armitage trend test. Data management and analysis were performed using SPSS 25.0 software (IBM Corporation, 2020, USA) or R version 4.2.0 programming language (www.r-project.org). All p-values were two-tailed, and a p-value below 0.05 was considered statistically significant.

### GWAS summary statistics for two-sample MR study

After conducting a comprehensive search in publicly available resources, we obtained GWAS summary statistics of 13 liver enzymes or proteins from the East Asian population to be used as exposures. These include total bilirubin (TBIL, bbj-a-53), total protein (TP, bbj-a-56), albumin (ALB, bbj-a-9), globulin protein (GLO, bbj-a-42), ALB/GLO ratio (A/G, bbj-a-4), alanine aminotransferase (ALT, bbj-a-6), aspartate aminotransferase (AST, bbj-a-8), gamma glutamyl transferase (GGT, bbj-a-23), alkaline phosphatase (ALP, bbj-a-5), lactate dehydrogenase (LDH, bbj-a-30), and glucose in blood (GLU, bbj-a-10) from the BioBank Japan (BBJ) project. Additionally, we obtained GWAS summary statistics for direct bilirubin (DBIL, ukb-e-30660_EAS) and indirect bilirubin (IBIL, ukb-e-recode1_EAS) from the MRC Integrative Epidemiology Unit (IEU) project (https://gwas.mrcieu.ac.uk/), and the summary statistics were derived exclusively from an East Asian cohort of UK Biobank database. Moreover, we collected GWAS summary statistics for PLT count in the Asian population from the BBJ project, which served as the target outcome (bbj-a-49).

The BBJ project is the first patient-based biobank in Japan, with approximately 200,000 participants recruited from 12 medical institutions between 2003 and 2008 (29). This project encompasses 47 types of diseases and phenotypes. For the 11 types of liver enzymes/proteins and PLT count, the project obtained laboratory measurement results from around 100,000 subjects who did not have hepatitis B or C virus, cirrhosis, hepatocellular carcinoma, rheumatoid arthritis, nephrotic syndrome, or hematopoietic tumor. In addition, the BBJ project conducted whole-genome sequencing assays and genotyped 8,885,115 SNPs for all the participants. For the DBIL and IBIL measurements, this project obtained measurement results from around 2,200 subjects and genotyped information from approximately 8,264,000 SNPs. Detailed information regarding GWAS summary statistics of the 13 liver enzymes/proteins and PLT count can be found in **Supplementary Table S2**.

### Two-sample MR study design and analysis

**Figure 1:**
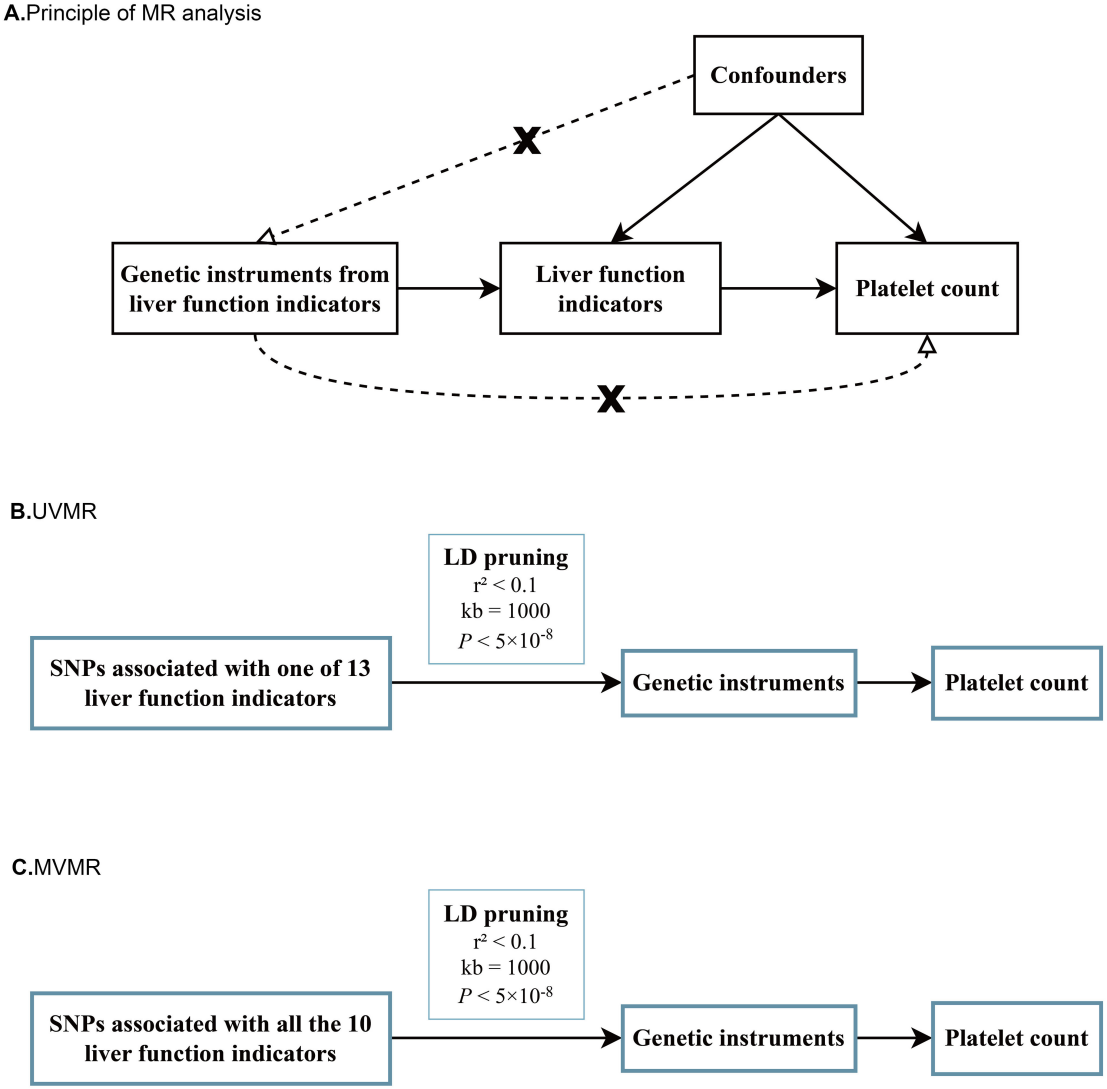
Schematic diagrams for principal of MR analyses, UVMR and MVMR. **(A)** Schematic diagram for exploring potential causal effects of various liver function indicators on platelet count; **(B)** Schematic diagrams for univariate MR analyses of 13 liver function indicators on platelet count; **(C)** Schematic diagrams of multivariate MR analyses for 10 liver function indicators on platelet count at one time.

The primary MR analyses consisted of two sequential steps. In the first step, we performed univariate MR (UVMR) analysis to assess whether each liver enzyme/protein had a genetic impact on the PLT count, which directly indicates the risk of thrombocytopenia (**Figures 1A, B**). Each liver enzyme/protein was considered as an exposure and the PLT count was treated as an outcome in these analyses. The main purpose was to determine if there was a causal relationship between individual liver enzymes/proteins and the level of PLT count. Subsequently, we conducted multivariate MR (MVMR) analyses to identify liver enzymes/proteins that had independent genetic effect on the PLT count, while accounting for the influence of other dependent factors (**Figure 1C**). This approach helped us avoid potential bias arising from the interaction between multiple exposures and the outcome, as liver enzymes or proteins are known to closely interact with each other (30–32).

Prior to conducting the MR analyses, all GWAS summary statistics underwent a series of preprocessing steps. These steps included the addition of missing but necessary parameters and the removal of strongly correlated SNPs based on pre-setting criteria (*r* ^2^<0.1, distance=1000 kb). Detailed information can be found in the **Supplementary Materials-Methods** section. Following preprocessing, independent SNPs with an original p-value below 5×10–^8^ were considered as valid instrumental variables. The strength of all valid variables was assessed to be strong or weak using the widely utilized formula F= beta^2^/se^2^ (33). All two-sample MR analyses were conducted using the ‘TwoSampleMR’ package (version 0.4.26) in the R programming language (version 4.2.0).

In contrast to previous common applications, we employed the most suitable MR method for each analysis scenario to reduce potential bias from method misapplication, as demonstrated in our prior work (34, 35). Detailed information of the most suitable MR method selection can be found in **Supplementary Materials-Methods** section. By selecting the most suitable MR method, we tested a single hypothesis for each pair of exposure and outcome, considering a p-value below 0.05 as indicative of significant genetic correlations. Additionally, we applied the false discovery rate (FDR) to adjust for multiple corrections and mitigate potential false positive signals (36). Regarding the effect size of causal correlation when the outcome was a continuous trait, we interpreted it as the average changes in the outcome per unit increase in the exposure. Furthermore, we employed the Steiger directionality test to evaluate the validity of the causal directions between the hypothesized exposures and outcomes (17, 37–40).

## Results

### Baseline characteristics of participants in clinical observational study

The basic demographic and clinical characteristics of the 16,464 participants were presented in **Table 1**. Among them, 2,730 (16.58%) individuals tested positive for HBsAg. HBsAg-positive subjects tended to be older (42.92 ± 9.16 *vs.* 40.95 ± 11.04, *P* < 0.001), predominantly male (59.56% *vs.* 48.90%, *P* < 0.001), and had higher FIB-4 values (0.94 ± 0.42 *vs.* 0.78 ± 0.36, *P* < 0.001) compared to healthy controls. HBsAg-positive subjects also had lower levels of WBC, NEU, LYMPH, RBC, Hb and ALB, but higher levels of DBIL, ALB/GLO, ALT, AST, GGT and GLU compared to healthy controls. Notably, the mean PLT count was significantly lower in HBsAg-positive subjects (230.91 ± 52.92 *vs.* 256.11 ± 57.55, *P* < 0.001), and the prevalence of thrombocytopenia (defined as PLT < 150×10^9^/L) was significantly higher in HBsAg-positive subjects compared to healthy controls (4.79% *vs.* 1.52%, *P*< 0.001).

**Table 1:** Basic demographic and clinical characteristics of the 16,464 included participants.

Characteristics	Overall	HBsAg(-)	HBsAg(+)	*P*
Participants	16,464	13,734 (83.42%)	2730 (16.58%)	
Sex				**<0.001**
Male	8,342 (50.67%)	6,716 (48.9%)	1,626 (59.6%)	
Female	8,122 (49.33%)	7,018 (51.1%)	1,104 (40.4%)	
Age (years)	41.27 ± 10.77	40.95 ± 11.04	42.92 ± 9.16	**<0.001**
HBsAb				**<0.001**
Positive	11,025 (66.96%)	11,025 (80.3%)	0 (0%)	
Negative	5,439 (33.04%)	2,709 (19.7%)	2730 (100%)	
HBeAg				**<0.001**
Positive	318 (1.93%)	0 (0%)	318 (11.6%)	
Negative	16,146 (98.07%)	13,734 (100%)	2,412 (88.4%)	
HBeAb				**<0.001**
Positive	4,416 (26.82%)	2,146 (15.6%)	2,270 (83.2%)	
Negative	12,048 (73.18%)	11,588 (84.4%)	460 (16.8%)	
HBcAb				**<0.001**
Positive	10,621 (64.51%)	7,895 (57.5%)	2,726 (99.9%)	
Negative	5,843 (35.49%)	5,839 (42.5%)	4 (0.1%)	
WBC (10^9^/L)	6.30 ± 1.62	6.33 ± 1.62	6.11 ± 1.63	**<0.001**
NEU (10^9^/L)	3.58 ± 1.22	3.61 ± 1.23	3.43 ± 1.19	**<0.001**
LYMPH (10^9^/L)	2.06 ± 0.61	2.07 ± 0.58	2.03 ± 0.75	**0.006**
MONO (10^9^/L)	0.34 ± 0.11	0.34 ± 0.11	0.34 ± 0.11	0.171
RBC (10^12^/L)	4.78 ± 0.49	4.77 ± 0.49	4.82 ± 0.49	**<0.001**
Hb (g/L)	145.03 ± 16.06	144.49 ± 16.05	147.74 ± 15.88	**<0.001**
PLT (10^9^/L)	251.94 ± 57.58	256.11 ± 57.55	230.91 ± 52.92	**<0.001**
Thrombocytopenia				**<0.001**
Yes	340 (2.07%)	209 (1.5%)	131 (4.8%)	
No	16,124 (97.93%)	13,525 (98.5%)	2,599 (95.2%)	
FIB-4	0.81 ± 0.37	0.78 ± 0.36	0.94 ± 0.42	**<0.001**
TBIL (μmol/L)				0.236
< 26	15,810 (96.03%)	13,200 (96.1%)	2,610 (95.6%)	
≥ 26	654 (3.97%)	534 (3.9%)	120 (4.4%)	
DBIL (μmol/L)				**<0.001**
< 8	15,707 (95.40%)	13,137 (95.7%)	2,570 (94.1%)	
≥ 8	757 (4.60%)	597 (4.3%)	160 (5.9%)	
IBIL (μmol/L)				0.278
<19	15,914 (96.66%)	2,629 (96.3%)	13,285 (96.7%)	
≥19	550 (3.34%)	101 (3.7%)	449 (3.3%)	
TP (g/L)				0.387
< 65	257 (1.56%)	220 (1.6%)	37 (1.4%)	
≥ 65	16,207 (98.44%)	13,514 (98.4%)	2,693 (98.6%)	
ALB (g/L)				**0.017**
< 40	86 (0.52%)	63 (0.5%)	23 (0.8%)	
≥ 40	16,378 (99.48%)	13,671 (99.5%)	2,707 (99.2%)	
GLO (g/L)				1
< 40	16,455 (99.95%)	2,729 (99.96%)	13,726 (99.9%)	
≥ 40	9 (0.05%)	1 (0.04%)	8 (0.1%)	
ALB/GLO				**<0.001**
< 1.2	1,689 (10.26%)	1,315 (9.6%)	374 (13.7%)	
≥ 1.2	14,775 (89.74%)	12,419 (90.4%)	2,356 (86.3%)	
ALT (U/L)				**<0.001**
< 40	14,374 (87.31%)	12,120 (88.2%)	2,254 (82.6%)	
≥ 40	2,090 (12.69%)	1,614 (11.8%)	476 (17.4%)	
AST (U/L)				**<0.001**
< 40	15,832 (96.16%)	13,278 (96.7%)	2,554 (93.6%)	
≥ 40	632 (3.84%)	456 (3.3%)	176 (6.4%)	
ALT/AST				**<0.001**
< 1	8,094 (49.16%)	7,043 (51.3%)	1,051 (38.5%)	
≥ 1	8,370 (50.84%)	6,691 (48.7%)	1,679 (61.5%)	
GGT (U/L)				**<0.001**
< 60	14,939 (90.74%)	12,412 (90.4%)	2,527 (92.6%)	
≥ 60	1,525 (9.26%)	1,322 (9.6%)	203 (7.4%)	
ALP (U/L)				0.569
< 125	16,262 (98.77%)	13,562 (98.7%)	2,700 (98.9%)	
≥ 125	202 (1.23%)	172 (1.3%)	30 (1.1%)	
LDH (U/L)				0.213
< 250	15,939 (96.81%)	13,307 (96.9%)	2,632 (96.4%)	
≥ 250	525 (3.19%)	427 (3.1%)	98 (3.6%)	
GLU (mmol/L)				**0.016**
<6.1	15,691(95.30%)	13,114(95.5%)	2,577(94.4%)	
≥6.1	773(4.70%)	620(4.5%)	153(5.6%)	

Thrombocytopenia was defined as PLT < 150×10^9^/L. P-values below 0.05 are in bold.

### Independent risk factors for thrombocytopenia in HBsAg positive subjects

The characteristics of participants with and without thrombocytopenia in 2,730 HBsAg-positive subjects were displayed in **Supplementary Table S1**. Individuals with thrombocytopenia had lower levels of ALB but higher levels of Age, FIB-4, TBIL, DBIL, ALT, AST and ALP. Since only 131 (4.80%) patients were diagnosed with thrombocytopenia and had older ages (47.04 ± 8.43 *vs.* 42.71 ± 9.15, *P*<0.001), propensity score matching (PSM) was utilized to adjust for baseline age and reduce the impact of selection bias between the thrombocytopenia and non-thrombocytopenia groups. After PSM, baseline age was balanced between the two groups (47.04 ± 8.43 *vs.* 47.04 ± 8.40, *P*=0.997), as shown in **Table 2**. Variables such as WBC, NEU, LYMPH, MONO, RBC and Hb were excluded from univariate logistic regression analysis as they were not considered casual factors of PLT based on the current understanding of hematogenous mechanism. Additionally, FIB-4 was not included in logistic regression analysis to avoid potential multicollinearity issue since it could be calculated from age, ALT, AST, and PLT count. Variables with a p-value below 0.1 in univariate logistic regression analysis were included in the subsequent multivariate logistic regression. Stepwise multiple logistic regression analyses identified independent factors significantly associated with thrombocytopenia risk in HBsAg positive subjects, including lower ALB, higher TBIL, higher ALT, and higher ALP, with corresponding ORs (95% CI) of 6.51 (1.32-34.83), 3.71 (1.85-7.24), 1.95 (1.21-3.10), and 6.60 (1.77-26.99), respectively, as shown in **Table 3**; **Supplementary Figure S2**.

**Table 2:** Basic demographic and clinical characteristics of the 131 HBsAg-positive subjects and 655 matched controls after propensity score matching.

PSM Characteristics	Platelet count		*P*
	< 150×10^9^/L	≥ 150×10^9^/L	
No. of subjects	131	655	
Sex			0.871
Male	79 (60.3%)	387 (59.1%)	
Female	52 (39.7%)	268 (40.9%)	
Age (years)	47.04 ± 8.43	47.04 ± 8.40	0.997
HBsAg			1
Positive	131 (100%)	655 (100%)	
Negative	0 (0%)	0 (0%)	
HBsAb			1
Positive	0 (0%)	0 (0%)	
Negative	131 (100%)	655 (100%)	
HBeAg			0.328
Positive	16 (12.21%)	59 (9.01%)	
Negative	115 (87.79%)	596 (91.99%)	
HBeAb			0.341
Positive	108 (82.44%)	564 (86.11%)	
Negative	23 (17.56%)	91 (13.89%)	
HBcAb			1
Positive	131 (100%)	654 (99.85%)	
Negative	0 (0%)	1 (0.15%)	
WBC (10^9^/L)	5.08 ± 1.31	6.22 ± 1.62	**<0.001**
NEU (10^9^/L)	2.79 ± 0.99	3.58 ± 1.28	**<0.001**
LYMPH (10^9^/L)	1.74 ± 0.45	1.99 ± 0.57	**<0.001**
MONO (10^9^/L)	0.29 ± 0.10	0.35 ± 0.12	**<0.001**
RBC (10^12^/L)	4.70 ± 0.59	4.79 ± 0.48	0.66
Hb (g/L)	146.04 ± 17.56	147.16 ± 14.90	0.493
PLT (10^9^/L)	131.08 ± 13.18	235.91 ± 50.15	**<0.001**
TBIL (μmol/L)			**0.001**
< 26	116 (88.5%)	630 (96.2%)	
≥ 26	15 (11.5%)	25 (3.8%)	
DBIL (μmol/L)			**0.002**
< 8	114 (87%)	621 (94.8%)	
≥ 8	17 (13%)	34 (5.2%)	
TP (g/L)			0.266
< 65	3 (2.3%)	5 (0.8%)	
≥ 65	128 (97.7%)	650 (99.2%)	
ALB (g/L)			**0.017**
< 40	4 (3.1%)	3 (0.5%)	
≥ 40	127 (96.9%)	652 (99.5%)	
ALB/GLO			0.239
< 1.2	22 (16.8%)	82 (12.5%)	
≥ 1.2	109 (83.2%)	573 (87.5%)	
ALT (U/L)			**0.006**
< 40	99 (75.6%)	561 (85.6%)	
≥ 40	32 (24.4%)	94 (14.4%)	
AST (U/L)			**0.029**
< 40	114 (87%)	610 (93.1%)	
≥ 40	17 (13%)	45 (6.9%)	
ALT/AST			0.922
< 1	51 (38.9%)	261 (39.8%)	
≥ 1	80 (61.1%)	394 (60.2%)	
GGT (U/L)			0.239
< 60	118 (90.1%)	612 (93.4%)	
≥ 60	13 (9.9%)	43 (6.6%)	
ALP (U/L)			**0.001**
< 125	125 (95.4%)	651 (99.4%)	
≥ 125	6 (4.6%)	4 (0.6%)	
LDH (U/L)			0.936
< 250	125 (95.4%)	629 (96.1%)	
≥ 250	6 (4.6%)	26 (3.9%)	
GLU (mmol/L)			0.669
<6.1	123(93.89%)	605(92.37%)	
≥6.1	8 (6.11%)	50 (7.63%)	

P-values below 0.05 are in bold.

**Table 3:** Univariate and multivariate logistic regression analysis of various liver function indicators with thrombocytopenia risk in HBsAg-positive subjects.

Variable	Univariate analysis		Multivariate analysis	
	OR (95%CI)	*P*	OR (95%CI)	*P*
Sex				
Female	reference			
Male	1.107(0.717-1.708)	0.646		
Age	1.000 (0.977-1.024)	0.998		
HBeAg				
Negative	reference			
Positive	1.199 (0.449-3.205)	0.718		
HBeAb				
Negative	reference			
Positive	0.911 (0.393-2.115 )	0.829		
HBcAb				
Negative	reference			
Positive	125057.4 (0-Inf)	0.983		
TBIL				
<26	reference		reference	
≥26	3.088 (1.073-8.882)	**0.037**	3.71(1.85-7.24)	**<0.001**
DBIL				
<8	reference			
≥8	1.338(0.495-3.614)	0.566		
TP				
<65	reference			
≥65	0.498 (0.091-2.715)	0.421		
ALB				
≥40	reference		reference	
<40	5.188 (0.91-29.564)	**0.064**	6.51(1.32-34.83)	**0.019**
ALB/GLO				
< 1.2	reference			
≥1.2	0.878(0.497-1.553)	0.656		
ALT				
<40	reference		reference	
≥40	1.998 (1.026-3.89)	**0.042**	1.95(1.21-3.10)	**0.005**
AST				
<40	reference			
≥40	1.151(0.512-2.585)	0.734		
ALT/AST				
< 1	reference			
≥1	0.81(0.514-1.277)	0.365		
GGT				
<60	reference			
≥60	1.046 (0.488-2.244)	0.908		
ALP				
<125	reference		reference	
≥125	6.545(1.696-25.259)	**0.006**	6.60 (1.77-26.99)	**0.005**
LDH				
<250	reference			
≥250	0.853(0.33-2.206)	0.743		
GLU				
<6.1	reference			
≥6.1	0.583(0.242-1.406)	0.230		

P-values below 0.1 in univariate analysis are in bold. P-values below 0.05 in multivariate analysis are in bold.

### Selection of valid genetic variants for MR analyses

We initially selected suitable independent genetic instruments from various exposure GWAS datasets based on the predetermined criteria (*r* ^2^<0.1, distance=1000 kb, *P*<5×10^-8^). In our series of MR analyses, we considered 13 different liver function indicators as exposures. Independent genetic variants were chosen from 13 GWAS datasets corresponding to GLU (n=24), GGT (n=130), LDH (n=55), A/G (n=91), GLO (n=115), ALP (n=115), TBIL (n=70), TP (n=65), ALT (n=48), AST (n=59), ALB (n=25), DBIL (n=3), and IBIL (n=7). The specific numbers of independent SNPs and significant signals used in the subsequent MR analyses were presented in **Table 4**; **Supplementary Table S2**.

**Table 4:** Univariable MR analyses with strong genetic instruments (*P*<5×10^-8^) for causal effects of 13 liver function indicators on platelet count.

Exposure	Outcome	No. of clumped SNPs ^a^	No. of SNPs in MRA ^b^	MR *adjusted P* ^c^	beta(se)	Most suitable MR Method ^d^	Heterogeneity	Pleiotropy	Directionality
**ALT**	PLT	48	45	**1.70×10^-5^**	-0.41(0.09)	IVW(random effects)	Yes	No	True
**GGT**		130	129	**1.73×10^-3^**	-0.13(0.03)	IVW(random effects)	Yes	No	True
**TP**		65	61	**2.70×10^-3^**	0.11(0.03)	Weighted median	Yes	No	True
**AST**		59	58	**2.80×10^-3^**	-0.32(0.10)	IVW(random effects)	Yes	No	True
**A/G**		91	88	**2.80×10^-3^**	-0.09(0.03)	IVW(random effects)	Yes	No	True
**GLU**		24	21	**5.88×10^-3^**	0.13(0.04)	Weighted median	Yes	No	True
**GLO**		115	113	**6.55×10^-3^**	0.06(0.02)	IVW(random effects)	Yes	No	True
ALP		115	114	6.08×10^-2^	0.02(0.01)	IVW(random effects)	Yes	No	True
ALB		25	25	1.01×10^-1^	0.14(0.08)	IVW(random effects)	Yes	No	True
IBIL		7	4	1.01×10^-1^	-0.01(0.01)	IVW (fixed effects)	No	No	True
DBIL		3	3	3.48×10^-1^	-0.01(0.01)	IVW (fixed effects)	No	No	True
TBIL		70	70	6.03×10^-1^	0.01(0.02)	MR Egger	Yes	Yes	True
LDH		55	54	9.57×10^-1^	0.00(0.02)	IVW(random effects)	Yes	No	True

^a^No. of clumped SNPs: number of independent genetic SNPs with a p-value <5×10–^8^ after clumping; ^b^No. of SNPs in MRA: number of independent genetic SNPs used in the MR analysis for each pair of exposure and outcome; ^c^MR adjusted *P*: adjusted p-value of the most suitable MR method; Adjusted p-values below 0.05 are in bold; ^d^the most suitable MR method used in MR analysis.

### Genetic causal effects of liver function indicators on thrombocytopenia risk

**Figure 2:**
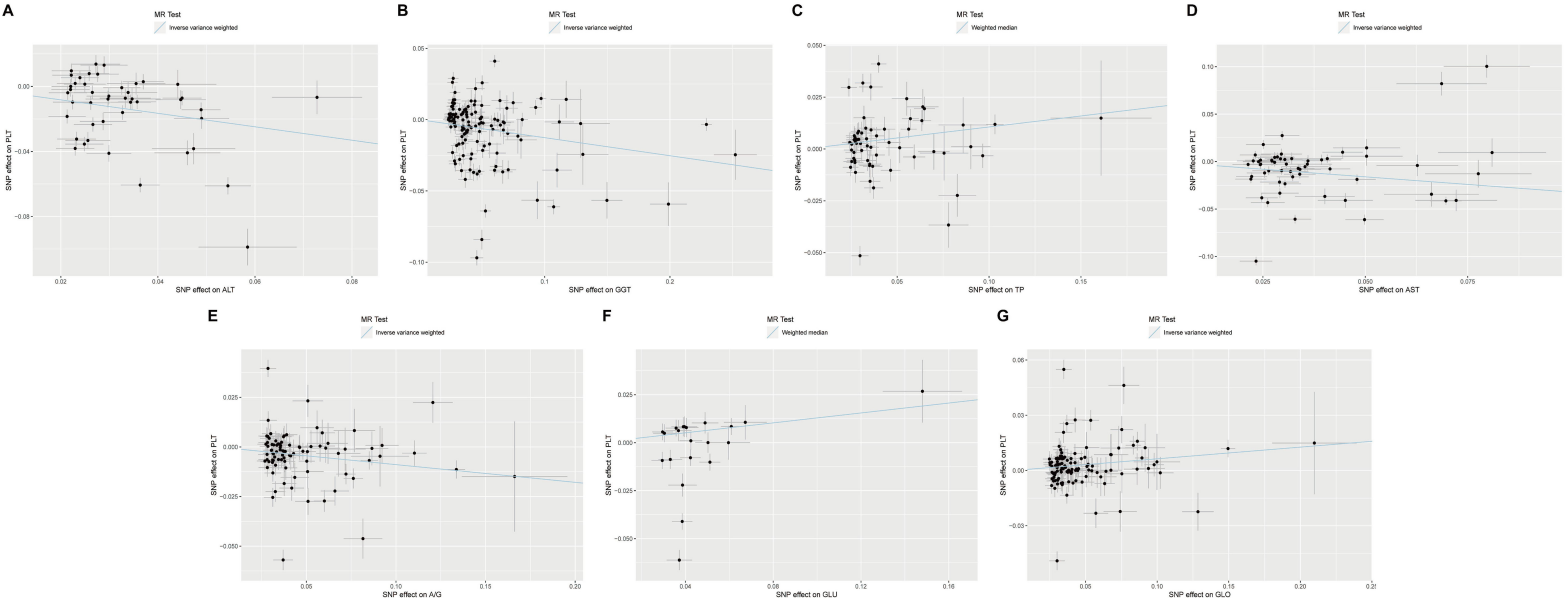
Scatter plots showing significant causal effects of seven liver function indicators on platelet count with the most suitable MR method in UVMR. **(A–G)** Significant causal effects of ALT, GGT, TP, AST, A/G, GLU and GLO on platelet count in UVMR, respectively. UVMR: univariate Mendelian randomization.

To investigate the potential causal effect of each liver enzyme/protein on the risk of thrombocytopenia, we treated each liver enzyme/protein as an exposure and the PLT count as the outcome. Using the most appropriate MR method, our analyses revealed that multiple liver enzyme/proteins had a significant causal effect on the PLT count. Specifically, we observed that ALT (β=-0.41, FDR=1.70×10^-5^), GGT (β=-0.13, FDR=1.73×10^-3^), TP (β=0.11, FDR=2.70×10^-3^), AST (β=-0.32, FDR=2.80×10^-3^), A/G (β=-0.09, FDR=2.80×10^-3^), GLU (β=0.13, FDR=5.88×10^-3^), and GLO (β=0.06, FDR=6.55×10^-3^) were all found to causally affect the PLT count (**Table 4**, **Figures 2A-G**). Notably, the acting directions of the significant signals in the UVMR analyses were consistent with the true direction of causality (**Table 4**). However, it is worth noting that in the UVMR analysis, the absolute value of effect size was employed to gauge the strength of causal correlations, as one indicator’s effect might be influenced by another within the UVMR analysis framework. Subsequently, we conducted a set of multivariate MR (MVMR) analyses to identify the independent causally correlated liver function indicators. Since TP and A/G data could be directly calculated from ALB and GLO data, they were excluded from the follow-up MVMR analyses (**Table 5**). Additionally, TBIL data was also removed as it could be calculated from DBIL and IBIL data. In this set of MVMR analyses, we found that the GLO protein exhibited a positive causal correlation with the PLT count (β=0.10, FDR=2.08×10^-2^) (**Table 5**; **Supplementary Figure S3A**). Furthermore, GLU, AST, and ALT were inversely causally correlated with the PLT count, respectively (β=-0.15, FDR=2.73×10^-2^; β=-0.21, FDR=2.73×10^-2^; β=-0.18, FDR=4.65×10^-2^) (**Table 5**; **Supplementary Figures S3B, C, E**). Interestingly, although the outcome was negative in UVMR, the ALB showed a marginal correlation with the PLT count in MVMR (**Supplementary Figure S3D**). These inconsistent results may be attributed to the strong relationship between the ALB, GLO, A/G, and TP. No other significant signals were identified in the MVMR analysis (**Table 5**).

**Table 5:** Multivariable MR analyses with strong genetic instruments (*P*<5×10^-8^) for causal effects of 10 liver function indicators on platelet count.

Exposure	Outcome	No. of clumped SNPs ^a^	No. of SNPs in MVMR ^b^	MVMR *adjusted P* ^c^	beta(se)
**GLO**	PLT	499	438	**2.08×10^-2^**	0.10(0.03)
**GLU**				**2.73×10^-2^**	-0.15(0.06)
**AST**				**2.73×10^-2^**	-0.21(0.08)
**ALB**				**4.47×10^-2^**	0.13(0.06)
**ALT**				**4.65×10^-2^**	-0.18(0.08)
ALP				1.29×10^-1^	0.03(0.02)
IBIL				5.57×10^-1^	-0.02(0.03)
GGT				7.27×10^-1^	-0.01(0.03)
LDH				7.27×10^-1^	0.02(0.03)
DBIL				8.63×10^-1^	0.00(0.03)

^a^No. of clumped SNPs: number of independent genetic SNPs with a p-value <5×10–^8^ after clumping; ^b^ No. of SNPs in MVMR: number of independent genetic SNPs used in the MVMR analysis for exposure and outcome; ^c^ MVMR adjusted *P*: adjusted p-value of the most suitable MVMR method; Adjusted p-values below 0.05 are in bold.

### Correlation of ALT categories with the prevalence of thrombocytopenia

**Figure 3:**
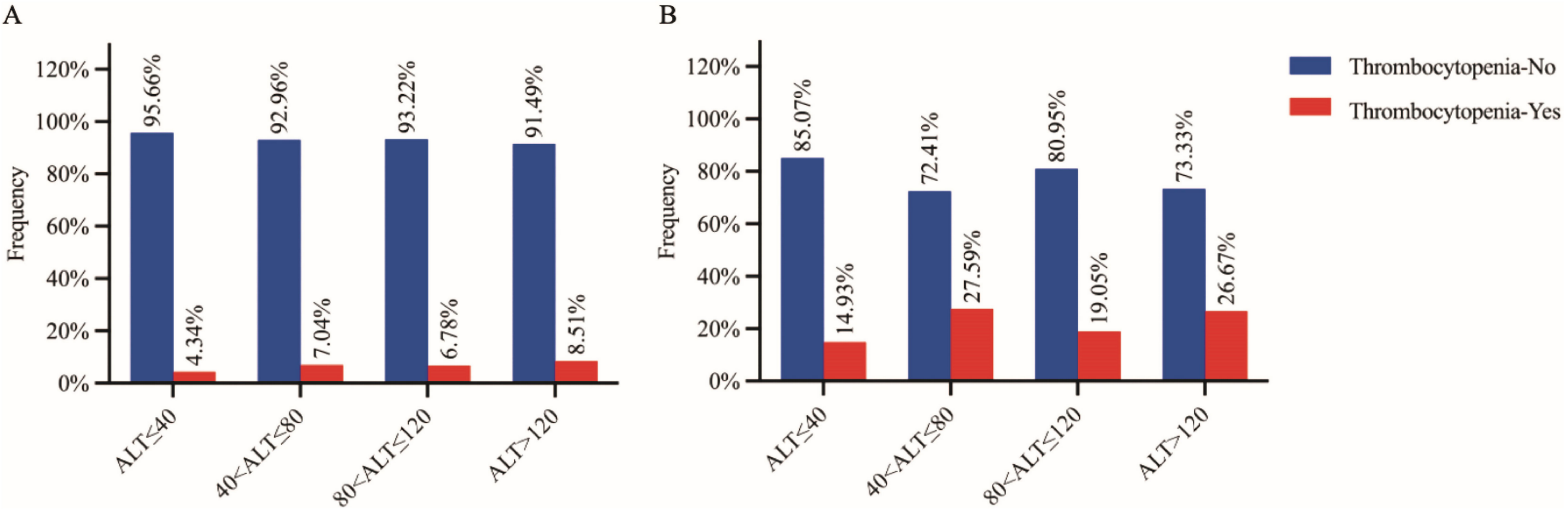
Proportion of thrombocytopenia subjects in HBsAg positive subjects (**A**: raw cohort and **B**: PSM cohort) grouped by four stratified alanine aminotransferase (ALT) levels. Thrombocytopenia-Yes/No: subjects with/without thrombocytopenia.

Since both clinical observational study and Mendelian randomization analysis suggested that ALT was a causal risk factor for the risk of thrombocytopenia, we further analyzed the relationship between ALT categories and the prevalence of thrombocytopenia. The initial ALT levels were categorized into four successive levels based on the reference interval: levels 1 (≤40 U/L), levels 2 (40–80 U/L), levels 3 (80–120 U/L), and levels 4 (>120 U/L). We found that the frequencies of thrombocytopenia gradually increased with the elevation of ALT in both the raw cohort (4.34%, 7.04%, 6.78% and 8.51%, **Figure 3A**) and the PSM cohort (14.93%, 27.59%, 19.05% and 26.67%, **Figure 3B**). We also observed significant trends indicating a higher prevalence of thrombocytopenia with the elevated ALT levels in both the raw cohort and the PSM cohort (Z= -2.415, *P*= 0.015 and Z= -2.461, *P*= 0.014, respectively, chi-square for trend).

## Discussion

In this study of a large cohort of apparently healthy participants who underwent comprehensive health examination, we observed a significantly higher prevalence of thrombocytopenia in early-stage HBV infection subjects without liver cirrhosis or splenomegaly compared to healthy controls (4.79% *vs.* 1.52%). Our multivariate logistic regression analyses revealed that higher TBIL, lower ALB, higher ALT, and higher ALP were independent factors significantly associated with the risk of thrombocytopenia in HBV infection subjects. Furthermore, our UVMR and MVMR analyses showed significantly causal correlations between ALT and the PLT count, suggesting that the hepatic injury indicator ALT may have a causal effect on the risk of thrombocytopenia in HBV infection cases. The relationship between ALT and thrombocytopenia was further explored, and significant trends of a higher prevalence of thrombocytopenia with elevated ALT levels were observed in both the clinical raw and PSM cohorts, supporting the significant causal effect of the ALT on the risk of thrombocytopenia.

For several decades, ALT has been regarded as a marker of liver injury, including various etiologies such as viral hepatitis and fatty liver (41). Additionally, elevated ALT levels have been associated with an increased risk of developing cardiovascular disease, obesity, insulin resistance, metabolic syndrome, and type 2 diabetes (42, 43). The underlying mechanisms for these associations were attribute to the involvement of ALT in gluconeogenesis, amino acid synthesis, iron stores and regulation of other liver metabolism functions, such as fatty acid, glycerolipid, and bile acids metabolism (44–47). Furthermore, evidences from MR analyses suggest that ALT has causal correlations with cardiovascular disease (48) and insulin resistance/type 2 diabetes (49, 50). Accumulating evidence indicates that ALT enzymatic activity should not be solely considered as a marker of liver injury, but also as highly correlated with multiple extrahepatic diseases (42–50). Based on our observational study and MR analyses, it is reasonable to speculate that ALT is causally correlated with thrombocytopenia risk in early-stage HBV infection patients without cirrhosis. One potential underlying mechanism is that ALT may decrease the synthesis of TPO, leading to the development of thrombocytopenia (51). In addition, we also found that elevated serum ALT has been identified as an independent marker of systemic inflammation and increased oxidative stress (52). Besides that, excessive reactive oxygen species was able to trigger apoptosis and reduce the lifespan of platelets (53, 54). Nevertheless, our study provided an additional etiology of thrombocytopenia in the early stages of HBV infection patients.

Indeed, thrombocytopenia has been observed in various other conditions, such as HCV infection (11), acute autochthonous hepatitis E (55), bunyavirus infection (56), dengue virus infection (57), COVID-19 infection (58), and preeclampsia and hemolysis (59). In addition, severe fever with thrombocytopenia syndrome (SFTS) caused by bunyavirus infection is associated with an elevated ALT level (60), and ALT has been identified as an independent predictor of SFTS mortality (61). For the first time, the present study provided solid supports for the potential causality of ALT in the occurrence of thrombocytopenia, prior to the development of liver cirrhosis.

In addition to ALT, our observational study also identified TBIL, ALB, and ALP as independent factors significantly associated with the risk of thrombocytopenia. However, our UVMR and MVMR analyses identified AST, GLO, and GLU as causal factors for the risk of thrombocytopenia. The differences in findings may be attributed to the different principles of logistic regression analysis and MR analysis. Furthermore, the observational study utilized clinical data from apparently healthy participants in a local hospital, while the MR study analyzes GWAS summary statistics from two large biobanks with complex ethical background. Despite the similar genetic background of both groups, the different sources of data may contribute to contrasting results. It is important to note that conventional retrospective observational studies have inherent limitations, including the potential influence of reverse causation and residual confounding factors. In contrast, MR analysis has emerged as a promising tool for causal inference, particularly with the rapid development of large-scale GWAS. MR analysis is advantageous as it is less susceptible to potential biases of confounding factors and reverse causation (17–21). Regarding the role of glucose metabolism, it has been established that glucose is essential for platelet activation, thrombosis, platelet production, and clearance from the circulation, primarily due to dysfunctional Ca^2+^ signaling. Studies have shown that in the absence of glycolysis, platelet counts were significantly reduced (62, 63). These findings provided solid support for our MR results, suggesting that glucose may be a causal factor for the risk of thrombocytopenia. However, it is important to acknowledge that the relatively small number of subjects with abnormal liver function indicators limited our ability to confirm the categorical stratification of these indicators and thrombocytopenia. Therefore, further investigations, such as randomized controlled trials and more in-depth mechanism studies, are needed to establish the causal relationship between these indicators and the risk of thrombocytopenia.

## Limitations

The present study had several potential limitations. Firstly, we were unable to use HBsAg or HBV DNA levels as indicators of HBV replication status. Secondly, liver cirrhosis was not confirmed through histological on liver biopsy but instead diagnosed rigorously using ultrasonography assessment by experienced radiologists. Additionally, the FIB-4 score was calculated to exclude patients with possible cirrhosis or splenomegaly. Thirdly, the study did not provide comprehensive information on factors such as antiviral treatment or other related variables that could potentially impact liver enzymes/proteins levels or platelet count. To minimize the impact of confounding factors, we selected participants from the Physical Examination Center, where most individuals are generally healthy. Severe CHB patients typically visit the outpatient department for medical treatment in our hospital. Therefore, the medication use rate among participants included in this study is low, and the impact of medication on PLT levels is minimal. We also excluded participants who were aged ≥60 years, had a FIB-4 score ≥3.25, had cirrhosis confirmed by ultrasonography, or were seropositive for both HBsAg and HBV surface antibody. These individuals are more likely to have a longer duration of HBV infection, receive medical therapy, and have a poorer health condition, which may affect liver enzyme levels and platelet counts. Finally, observational analyses are susceptible to residual confounding factors (such as lifestyle factors and socioeconomic status) and reverse causality, which pose challenges in establish causal relationship. Given that, we conducted a series of MR analyses to further validate the causally correlated liver injury indicator ALT. The MR analyses leverage germline genetic variants as instrumental variables to enable causal inference between a pair of exposure and outcome (64, 65).

## Conclusions

In a large cohort of apparently healthy participants, we observed a higher prevalence of thrombocytopenia in patients with early-stage HBV infection. In addition, our clinical observational study and MR analyses indicated significant correlations between multiple hepatic function indicators and the risk of thrombocytopenia, particularly for the hepatic injury biomarker ALT. Elevated level of ALT were consistently associated with a higher prevalence of thrombocytopenia in both the clinical raw and PSM cohorts. Regarding the potential underlying biological mechanism, it is postulated that ALT may have the ability to reduce the synthesis of TPO, thereby increasing the risk of thrombocytopenia. These findings suggest that interventions targeting the hepatic injury indicator ALT, either through pharmacological or lifestyle approaches, may hold promise in preventing thrombocytopenia in the early-stage HBV infection.

## Data Availability

The original contributions presented in the study are included in the article/**Supplementary Material**. Further inquiries can be directed to the corresponding authors.

## Supplementary material

Supplementary Figure 1 Flow chart for Chinese Han population participants included in clinical observation study. PSM cohort: propensity score matching cohort.

Supplementary Figure 2 Forest plot of significant risk factors for thrombocytopenia in multivariate logistic regression analysis. N: number of subjects.

Supplementary Figure 3 Scatter plots showing significant causal effects of liver function indicators on platelet count in MVMR. **(A–E)** Significant causal effects of GLO, GLU, AST, ALB and ALT on platelet count in MVMR, respectively. MVMR: multivariate Mendelian randomization.
