# Supplementary material for: Causal relationship between hepatic function indicators and thrombocytopenia risk in early-stage hepatitis B virus infection: evidence from clinical observational studies and mendelian randomization analyses
Source: Front Immunol. 2025 May 29;16:1440317. doi: 10.3389/fimmu.2025.1440317 (PMC12158931; doi:10.3389/fimmu.2025.1440317)
Supplement: Supplementary Figure 1 — Flow chart for Chinese Han population participants included in clinical observation study. PSM cohort: propensity score matching cohort. [file DataSheet1.docx]

**Supplementary Materials and Methods**

*Pre-processing of GWAS summary statistics*

First, we employed the LiftOver program (University of California Santa Cruz, CA, USA) to transfer the genomic positions into Ensembl GRCh37 release 108 for all the GWAS summary statistics. Afterward, several missing but necessary parameters were calculated using different approaches. Summary data without beta or standard error values were calculated using equations (1) and (2), respectively. Besides, more than half of the summary datasets lacked data regarding the necessary non-effect alleles, which would be added based on the reference genome (GRCh37). After adding the missing parameters, we further removed SNPs with a strong genetic correlation in the linkage disequilibrium block (*r*^2^ <0.1, distance=1000 kb) using the PLINK program (version 1.90). The correlations between SNPs were derived from the 1000 Genomes European or eastern Asian samples. SNPs with an original *p*-value below 5×10^-8^ would be treated as valid instrumental variables.

| $beta=log(OR)$ (1) | |
| --- | --- |
| $\mathrm{se}=\frac{log(\mathrm{OR})}{qnorm(\frac{P}{2})}, se=\frac{\sqrt{\mathrm{beta}^{2}}}{qchisq(P)}$ (2) |  |

OR: odds ratio for effect allele in the summary data; *P*: original *p*-value from the GWAS analysis; se: standard error.

*Selection strategy of the most suitable MR method*

We initially utilized MR Egger regression analysis to assess the presence of horizontal pleiotropy in the valid genetic instruments, and the MR inverse-variance weighted (IVW) method to evaluate heterogeneity among multiple genetic instruments. After conducting these assessments, the most suitable MR approach was selected for each of the four different scenarios. The fixed-effect IVW method was applied when there was neither horizontal pleiotropy nor significant heterogeneity (scenario 1, S1). The random-effect IVW method was employed when there was obvious heterogeneity but no horizontal pleiotropy (S2). The MR Egger method or weighted median was applied when there was an obvious horizontal pleiotropy (S3). The Wald ratio method was employed when there was only one valid genetic instrument in MR analysis (S4).

*Choice of thresholds for multiple liver function indicators*

We selected the cutoff value mainly according to the WS/T 404-2012, Reference intervals for common clinical biochemistry tests of National Health Commission (http://www.nhc.gov.cn/wjw/s9492/wsbz.shtml). With that, the reference threshold for TBIL is ≤26 μmol/L (male), DBIL is ≤ 8 μmol/L, TP is 65 g/L, ALB is 40 g/L, GLO is 40 g/L, A/G is 1.2:1, ALT is 50 U/L (male), AST is 40 U/L (male), ALP is 125 U/L (male), LDH is 250 U/L. For these indicators, we chose the cutoff values primarily based on the upper reference intervals for males. This strategy was guided by several factors: the convenience of statistics, the ease of interpreting the results, the higher prevalence of male CHB patients in China, and the clinical significance of each variable.

ALT is the most direct indicator reflecting liver inflammation. Both domestic and international guidelines recommend that ALT with a higher level than the upper limit of normal value (ULN) is an indication for antiviral treatment. Currently, the ULN of ALT commonly used worldwide is 40 U/ L. In China, the ULN of ALT is 50 U/L for males and 40 U/L for females according to the WS/T 404-2012, as showed before. In this study, we chose 40 U/L as the cutoff value since we specially focus on the chronic HBV infected patients. The reference intervals for fasting plasma glucose (FPG) are based on the WHO 1999 diagnostic criteria, which defined the normal fasting blood glucose range as 3.9–6.1 mmol/L. Therefore, the GLU cutoff value in this study is selected as ≤6.1 mmol/L and a value above 6.1 means impaired fasting blood glucose.

**Supplementary Figures**

**
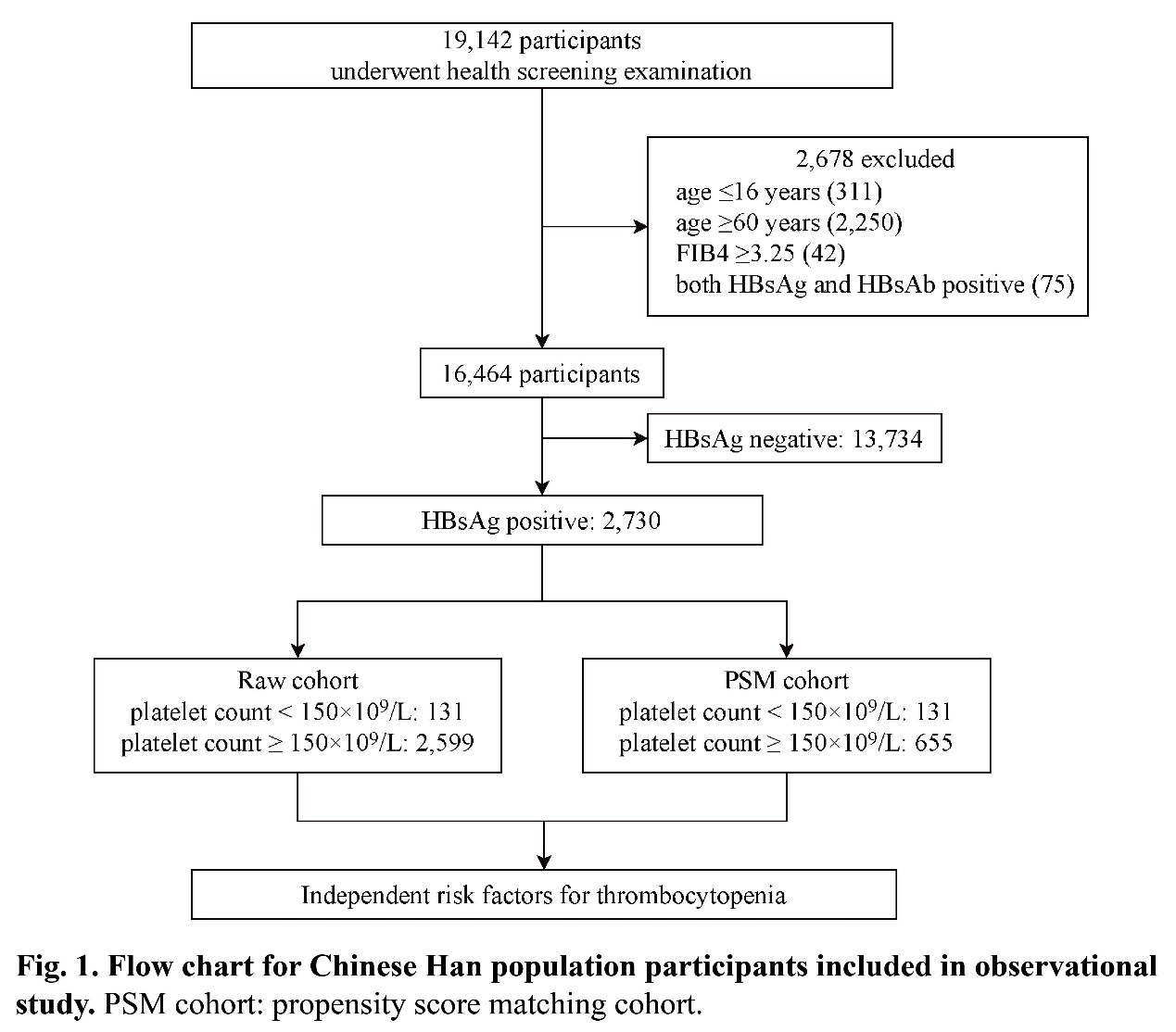
**

**Supplementary Figure 1. Flow chart for Chinese Han population participants included in clinical observation study.** PSM cohort: propensity score matching cohort.

**
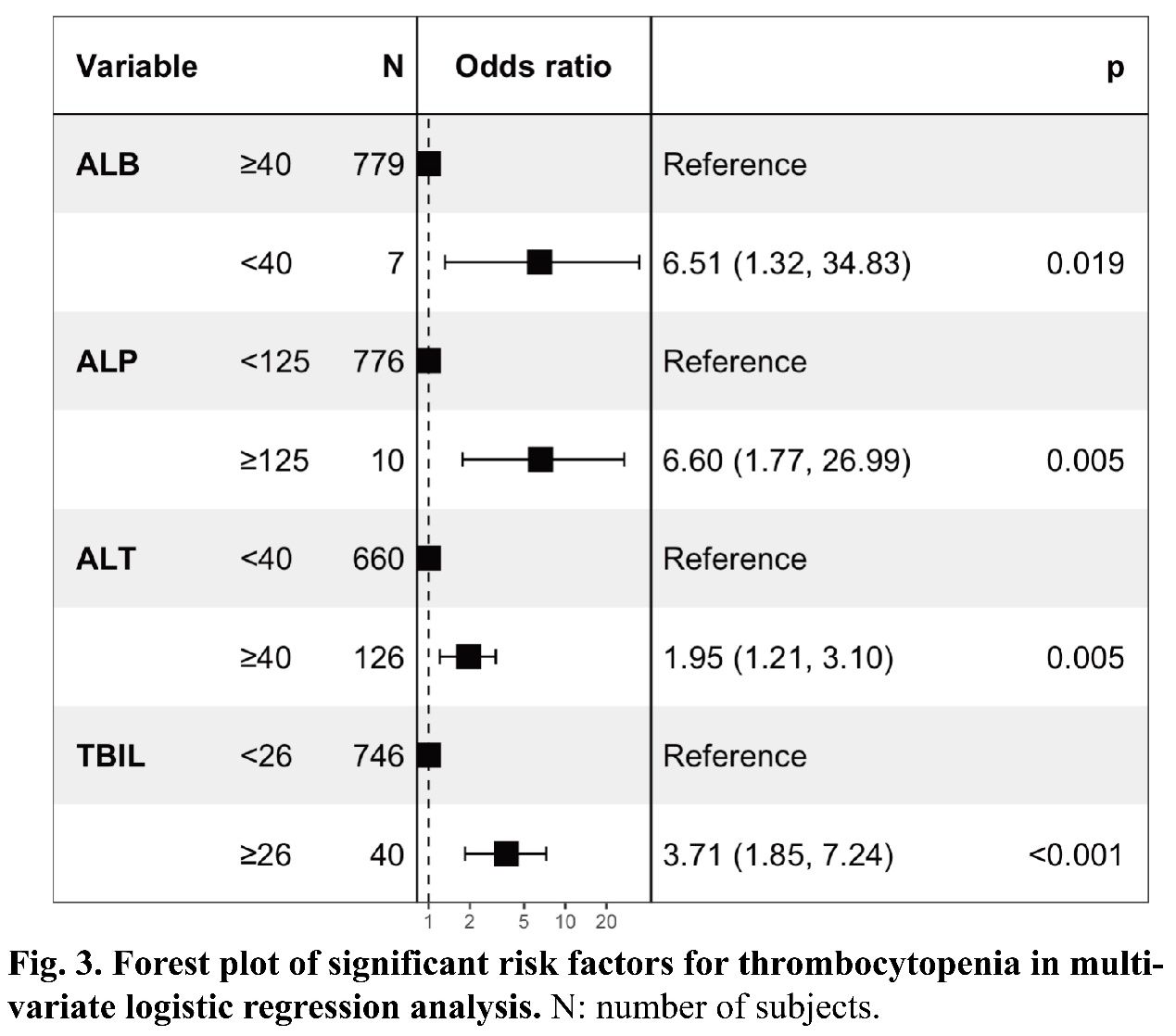
**

**Supplementary Figure 2. Forest plot of significant risk factors for thrombocytopenia in multivariate logistic regression analysis.** N: number of subjects.

**
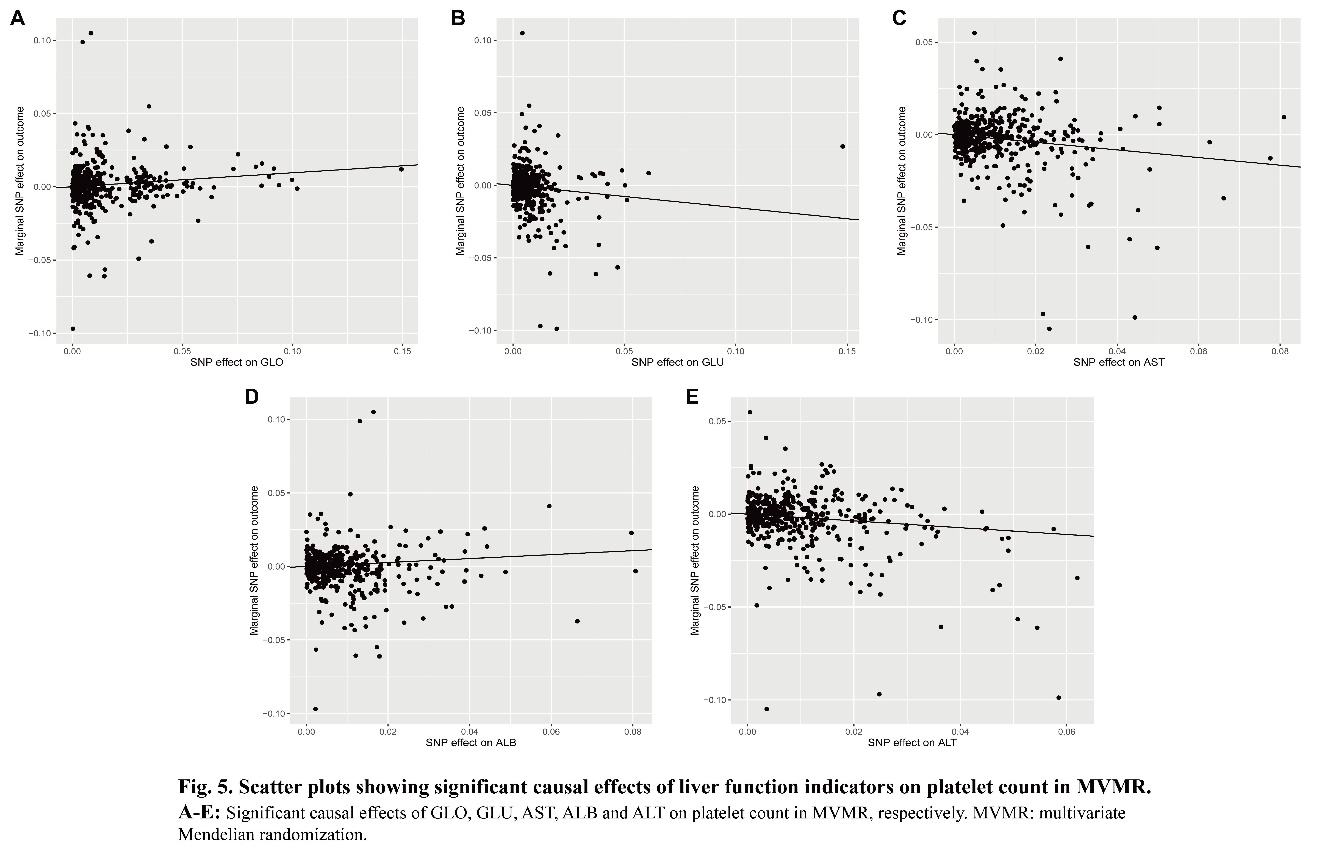
**

**Supplementary Figure 3. Scatter plots showing significant causal effects of liver function indicators on platelet count in MVMR.**

A-E: Significant causal effects of GLO, GLU, AST, ALB and ALT on platelet count in MVMR, respectively. MVMR: multivariate Mendelian randomization.

**Supplementary Tables**

**Supplementary Table 1.** Basic demographic and clinical characteristics of the 2,730 HBsAg-positive subjects.

| **﻿Raw Characteristics** | **﻿Platelet count** | | ***P*** |
| --- | --- | --- | --- |
|  | **< 150×10^9^/L** | **≥ 150×10^9^/L** |  |
| No. of subjects | 131 | 2,599 |  |
| Sex |  |  | 0.931 |
| male | 79 (60.3%) | 1,547 (59.5%) |  |
| female | 52 (39.7%) | 1,052 (40.5%) |  |
| Age (years) | 47.04 ± 8.43 | 42.71 ± 9.15 | **<0.001** |
| HBsAg |  |  | 1 |
| positive | 131 (100%) | 2,599 (100%) |  |
| negative | 0 (0%) | 0 (0%) |  |
| HBsAb |  |  | 1 |
| positive | 0 (0%) | 0 (0%) |  |
| negative | 131 (100%) | 2,599 (100%) |  |
| HBeAg |  |  | 0.946 |
| positive | 16 (12.2%) | 302 (11.6%) |  |
| negative | 115 (87.8%) | 2,297 (88.4%) |  |
| HBeAb |  |  | 0.919 |
| positive | 108 (82.4%) | 2,162 (83.2%) |  |
| negative | 23 (17.6%) | 437 (16.8%) |  |
| HBcAb |  |  | 1 |
| positive | 131 (100%) | 2,595 (99.8%) |  |
| negative | 0 (0%) | 4 (0.2%) |  |
| WBC (10^9^/L) | 5.08 ± 1.31 | 6.17 ± 1.63 | **<0.001** |
| NEU (10^9^/L) | 2.79 ± 0.99 | 3.46 ± 1.19 | **<0.001** |
| LYMPH (10^9^/L) | 1.74 ± 0.45 | 2.04 ± 0.76 | **<0.001** |
| MONO (10^9^/L) | 0.29 ± 0.10 | 0.34 ± 0.11 | **<0.001** |
| RBC (10^12^/L) | 4.70 ± 0.59 | 4.83 ± 0.49 | **0.004** |
| Hb (g/L) | 146.04 ± 17.56 | 147.83 ± 15.79 | 0.209 |
| PLT (10^9^/L) | 131.08 ± 13.18 | 235.94 ± 49.04 | **<0.001** |
| FIB-4 | 1.84 ± 0.54 | 0.89 ± 0.36 | **<0.001** |
| TBIL (μmol/L) |  |  | **<0.001** |
| < 26 | 116 (88.5%) | 2494 (96%) |  |
| ≥ 26 | 15 (11.5%) | 105 (4%) |  |
| DBIL (μmol/L) |  |  | **0.001** |
| < 8 | 114 (87%) | 2456 (94.5%) |  |
| ≥ 8 | 17 (13%) | 143 (5.5%) |  |
| TP (g/L) |  |  | 0.575 |
| < 65 | 3 (2.3%) | 34 (1.3%) |  |
| ≥ 65 | 128 (97.7%) | 2565 (98.7%) |  |
| ALB (g/L) |  |  | **0.019** |
| < 40 | 4 (3.1%) | 19 (0.7%) |  |
| ≥ 40 | 127 (96.9%) | 2580 (99.3%) |  |
| ALB/GLO |  |  | 0.355 |
| < 1.2 | 22 (16.8%) | 352 (13.5%) |  |
| ≥ 1.2 | 109 (83.2%) | 2247 (86.5%) |  |
| ALT (U/L) |  |  | **0.041** |
| < 40 | 99 (75.6%) | 2155 (82.9%) |  |
| ≥ 40 | 32 (24.4%) | 444 (17.1%) |  |
| AST (U/L) |  |  | **0.003** |
| < 40 | 114 (87%) | 2440 (93.9%) |  |
| ≥ 40 | 17 (13%) | 159 (6.1%) |  |
| ALT/AST |  |  | 0.99 |
| < 1 | 51 (38.9%) | 1000 (38.5%) |  |
| ≥ 1 | 80 (61.1%) | 1599 (61.5%) |  |
| GGT (U/L) |  |  | 0.346 |
| < 60 | 118 (90.1%) | 2409 (92.7%) |  |
| ≥ 60 | 13 (9.9%) | 190 (7.3%) |  |
| ALP (U/L) |  |  | **<0.001** |
| < 125 | 125 (95.4%) | 2575 (99.1%) |  |
| ≥ 125 | 6 (4.6%) | 24 (0.9%) |  |
| LDH (U/L) |  |  | 0.701 |
| < 250 | 125 (95.4%) | 2507 (96.5%) |  |
| ≥ 250 | 6 (4.6%) | 92 (3.5%) |  |
| GLU (mmol/L) |  |  | 0.951 |
| < 6.1 | 123 (93.9%) | 2454 (94.4%) |  |
| ≥ 6.1 | 8 (6.1%) | 145 (5.6%) |  |

P-values below 0.05 are in bold.

**Supplementary Table 2.** Basic characteristics of GWAS summary statistics for 13 liver function indicators and platelet count data.

| **Phenotype** | **Abbreviation** | **N** ^a^ | **No. of qualified SNPs** ^b^ | **No. of SNPs**  **(*P*< 5E-08)** | **PMID** |
| --- | --- | --- | --- | --- | --- |
| Glucose | GLU | 93,146 | 6,108,953 | 1,059 | 29403010 |
| Gamma glutamyl transferase | GGT | 118,309 | 6,108,953 | 6,304 |  |
| Lactate dehydrogenase | LDH | 126,319 | 6,108,953 | 1,890 |  |
| Albumin/globulin ratio | A/G | 98,626 | 6,108,953 | 3,622 |  |
| Globulin | GLO | 98,538 | 6,108,953 | 4,019 |  |
| Alkaline phosphatase | ALP | 105,030 | 6,108,953 | 4,206 |  |
| Total bilirubin | TBIL | 110,207 | 6,108,953 | 3,022 |  |
| Total protein | TP | 113,509 | 6,108,953 | 2,251 |  |
| Alanine aminotransferase | ALT | 134,182 | 6,108,953 | 2,538 |  |
| Aspartate aminotransferase | AST | 134,154 | 6,108,953 | 2,385 |  |
| Albumin | ALB | 102,223 | 6,108,953 | 768 |  |
| Direct bilirubin | DBIL | 2,159 | 8,264,378 | 260 | NA |
| Indirect bilirubin | IBIL | 2,165 | 8,263,516 | 283 | NA |
| Platelet count | PLT | 108,208 | 6,108,953 | 8,012 | 29403010 |

**^a^** N: sample size; **^b^** qualified SNPs: SNPs with all necessary data in autosomes are remained for further analyses. NA: not available.
